# Comparative efficacy of mind-body exercises on quality of life, anxiety, and depression in patients with breast cancer: a systematic review with pairwise and network meta-analysis of randomized controlled trials

**DOI:** 10.3389/fpubh.2026.1830453

**Published:** 2026-07-16

**Authors:** Tian Zhou, Yi Deng, Lin Li, Xiang Xue, Haijun Han, Ning Zhou

**Affiliations:** 1 School of Physical Education and Sports, Sichuan University, Chengdu, China; 2 Development and Planning Division, Sichuan University, Chengdu, Sichuan, China; 3 College of Physical Education and Health Sciences, Chengdu College of Arts and Sciences, Chengdu, China; 4 Youth League Committee of Sichuan University, Chengdu, China

**Keywords:** anxiety, breast cancer, depression, mind-body exercise, network meta-analysis, quality of life, systematic review

## Abstract

**Background:**

Comparative evidence on the effects of different mind-body exercises (MBEs) for psychological outcomes in breast cancer patients remains limited. This study aimed to compare the effects of Yoga, Tai Chi, Qigong, Pilates, and Dance on quality of life, anxiety, and depression in breast cancer patients.

**Methods:**

PubMed, Embase, Web of Science, and the Cochrane Library were searched from inception to January 31, 2026. Pairwise meta-analyses were performed using random-effects models with Hedges' g. Bayesian network meta-analyses were conducted to estimate standardized mean differences (SMDs) with 95% credible intervals (CrIs). Ranking probabilities were summarized using SUCRA values. Risk of bias and evidence certainty were assessed using the RoB 2 tool and GRADE framework, respectively.

**Results:**

Fifty-five RCTs involving 3,770 participants were included. Network meta-analysis showed that Qigong, Dance, and Yoga significantly improved quality of life compared with control, with Qigong showing the highest ranking probability (SUCRA 83.9%). For anxiety, Dance was significantly superior to control and showed the highest ranking probability (SUCRA 90.6%). For depression, Yoga was significantly superior to control and ranked highest, closely followed by Tai Chi (SUCRA 64.3% and 64.1%, respectively). No significant differences were detected between active interventions. Exercise frequency and total dose appeared to be consistent moderators across outcomes. Overall, 55% of included trials were judged to be at high risk of bias, and the certainty of evidence was low to very low.

**Conclusion:**

Mind-body exercises may improve quality of life, anxiety, and depression in breast cancer patients. However, because no significant differences were found between active interventions and the certainty of evidence was low to very low, SUCRA rankings should be interpreted as exploratory and hypothesis-generating rather than as evidence of definitive superiority. Higher exercise dose may be associated with greater improvements. Future high-quality head-to-head RCTs are needed to strengthen the evidence base.

**Systematic review registration:**

https://www.crd.york.ac.uk/PROSPERO/view/CRD420261335679, Identifier: CRD420261335679.

## 1 Introduction

Breast cancer remains the most commonly diagnosed malignancy among women worldwide. According to the Global Cancer Statistics 2022, female breast cancer accounted for approximately 11.6% of all new cancer cases, with an estimated 2.3 million new diagnoses annually (1). In the United States alone, breast cancer incidence has continued an upward trend, rising by 1% annually during 2012–2021, with an even steeper increase of 1.4% per year observed in women younger than 50 years (2). Despite significant advances in early detection and multimodal treatment strategies—including surgery, chemotherapy, radiotherapy, endocrine therapy, and targeted therapy—which have substantially improved survival rates, the 5-year relative survival rate now exceeding 90% in many developed countries (3), the diagnosis and treatment of breast cancer impose a profound psychological burden on patients throughout the cancer continuum.

Anxiety and depression are the two most prevalent psychological comorbidities among breast cancer patients. A systematic review and meta-analysis encompassing 282,203 patients reported that the prevalence of depression and anxiety among breast cancer patients was as high as 32.2% and 41.9%, respectively (4). A more recent global meta-analysis of 71 studies confirmed that the pooled prevalence of depression in women with breast cancer reached 30.2%, with considerable regional variation ranging from 8.3% to 83% across countries (5). These negative psychological states are not merely secondary symptoms; they have been demonstrated to significantly affect treatment compliance, immune function, and disease prognosis. Wang et al. (4) further revealed that depression was associated with increased cancer recurrence (HR = 1.24, 95% CI: 1.07–1.43), all-cause mortality (HR = 1.30, 95% CI: 1.23–1.36), and cancer-specific mortality (HR = 1.29, 95% CI: 1.11–1.49), while anxiety was similarly associated with elevated recurrence and all-cause mortality risk. Furthermore, the interplay between psychological distress and diminished health-related quality of life (HRQoL) creates a vicious cycle that may accelerate disease progression and hinder recovery (6). Therefore, effective management of anxiety, depression, and quality of life in breast cancer patients has become a critical priority in comprehensive oncology care.

Conventional pharmacological interventions, such as selective serotonin reuptake inhibitors (SSRIs) and benzodiazepines, although effective for managing anxiety and depression, are frequently accompanied by adverse effects including nausea, insomnia, sexual dysfunction, and potential drug interactions with oncological treatments (7). These limitations have prompted growing interest in non-pharmacological complementary and alternative therapies. The National Comprehensive Cancer Network (NCCN) Survivorship Guidelines recommend that cancer survivors engage in regular physical activity to improve physical functioning, reduce fatigue, and enhance psychological wellbeing (8). The American College of Sports Medicine (ACSM) Roundtable consensus statement further supports that combined moderate-intensity aerobic and resistance exercise performed two to three times per week for at least 12 weeks can significantly improve health-related quality of life, including reductions in fatigue and anxiety, both during and after cancer treatment (9).

Among the various forms of physical activity, mind-body exercises (MBEs) have gained considerable attention as a particularly promising intervention for cancer populations. Mind-body exercises, which integrate physical movements with mental concentration, breath regulation, and meditative awareness, encompass a diverse range of modalities including Tai Chi, Qigong (such as Baduanjin and Liuzijue), Yoga, Pilates, and Dance therapy (10). Unlike conventional aerobic or resistance exercise, MBEs emphasize the holistic integration of body, mind, and spirit, offering a low-to-moderate intensity approach that is more accessible and better suited to the physical condition of cancer patients (11). A growing body of randomized controlled trials (RCTs) has demonstrated that MBEs can effectively alleviate cancer-related fatigue, reduce anxiety and depression, improve sleep quality, and enhance overall quality of life in breast cancer patients (10, 12). For instance, Yoga has shown convincing evidence in reducing depressive symptoms (Hedges' G = −0.77, 95% CI: −0.93 to −0.61) in breast cancer patients (13), while Tai Chi has demonstrated superior efficacy in mitigating cancer-related fatigue and improving quality of life compared to several other exercise modalities (14).

However, despite the accumulating evidence supporting the beneficial effects of MBEs on breast cancer patients, several important issues warrant further investigation. Most existing systematic reviews have employed traditional pairwise meta-analysis, which is inherently limited to direct comparisons between two interventions at a time and cannot simultaneously evaluate multiple MBE modalities within a unified framework (15). Network meta-analysis (NMA), as an extension of conventional meta-analysis, enables the integration of both direct and indirect evidence to compare and rank multiple interventions simultaneously, thereby providing a more comprehensive evidence hierarchy for clinical decision-making (16). To date, however, four specific gaps remain. First, existing NMAs in this field have focused on single outcomes, such as depression (17) or quality of life and cancer-related fatigue (18), rather than simultaneously addressing the three most clinically relevant psychological outcomes (quality of life, anxiety, and depression) within a single analytic framework. Second, prior reviews have often grouped mind-body exercises with aerobic and resistance training under broad exercise classifications (19), which dilutes head-to-head comparisons among specific MBE modalities. Third, no prior synthesis has integrated pairwise and Bayesian network meta-analysis in a unified framework, an approach that anchors direct evidence while leveraging indirect evidence to rank modalities when head-to-head trials are sparse. Fourth, no prior MBE-specific NMA in breast cancer has formally examined intervention-dose parameters (frequency, duration, and total dose) as effect modifiers alongside the network estimates.

To address these gaps, the present study conducts a systematic review with both pairwise and Bayesian network meta-analysis of RCTs to compare five mind-body exercise modalities (Tai Chi, Qigong, Yoga, Pilates, and Dance) on quality of life, anxiety, and depression in breast cancer patients, and to examine intervention-dose parameters as effect modifiers. The findings are intended to support evidence-based, non-pharmacological supportive care strategies in breast cancer survivorship.

## 2 Methods

This systematic review and network meta-analysis was conducted in accordance with the Preferred Reporting Items for Systematic Reviews and Meta-Analyses for Network Meta-Analyses (PRISMA-NMA) 2015 extension statement (20). The study protocol was prospectively registered in the International Prospective Register of Systematic Reviews (PROSPERO) (registration number: CRD420261335679).

### 2.1 Search strategy

A comprehensive and systematic literature search was conducted across four electronic databases from their inception to January 31, 2026: PubMed, Embase, Web of Science, and the Cochrane Central Register of Controlled Trials (CENTRAL). The search was restricted to English-language publications. The search strategy was developed using a combination of subject headings (MeSH terms for PubMed and Cochrane, Emtree terms for Embase) and free-text keywords. The core search terms were structured around three conceptual blocks: (1) Population: “breast cancer,” “breast neoplasms”; (2) Intervention: “mind-body exercise,” “Tai Chi,” “Qigong,” “Baduanjin,” “Yoga,” “Pilates,” “dance therapy”; (3) Study design: “randomized controlled trial,” “random^*^.” Terms within each block were combined using the Boolean operator OR, and the three blocks were combined using AND. The search strategy was initially developed for PubMed and subsequently adapted for each database according to their specific indexing systems and syntax requirements. In addition, the reference lists of all included studies and relevant systematic reviews were manually screened to identify any additional eligible studies. The complete search strategies for all four databases are provided in Supplementary File 1.

### 2.2 Eligibility criteria

The eligibility criteria for this systematic review were established according to the PICOS framework (Population, Intervention, Comparator, Outcomes, and Study design), as summarized in Table 1.

**Table 1:** Eligibility criteria based on the PICOS framework.

Criteria	Description
Population	Patients diagnosed with breast cancer, including breast cancer survivors
Intervention	Any form of mind-body exercise, including but not limited to Tai Chi, Qigong (e.g., Baduanjin, Liuzijue), Yoga, Pilates, and Dance therapy, delivered alone or combined with usual care
Comparator	Any type of control condition, including usual care, waitlist, attention control, health education, sham exercise, or no intervention
Outcomes	At least one of the following measured by validated instruments: (1) quality of life (e.g., FACT-B, EORTC QLQ-C30, and SF-36); (2) anxiety (e.g., HADS-A, GAD-7, SAS, and STAI); (3) depression (e.g., HADS-D, PHQ-9, SDS, CES-D, and BDI)
Study design	Randomized controlled trials (RCTs), limited to English-language publications

### 2.3 Study selection

Study selection was performed independently by two reviewers in a two-stage process. In the first stage, duplicate records were removed using EndNote X21 software, and the remaining titles and abstracts were screened to exclude obviously irrelevant studies. In the second stage, full texts of potentially eligible studies were retrieved and assessed against the predefined eligibility criteria. The inter-rater agreement between the two reviewers was evaluated using the intraclass correlation coefficient (ICC), with values of < 0.50, 0.50–0.74, 0.75–0.89, and ≥0.90 interpreted as poor, moderate, good, and excellent agreement, respectively (21). Any disagreements were resolved through discussion or consultation with a third reviewer. The study selection process was documented and presented using the PRISMA flow diagram.

### 2.4 Data extraction

Data were independently extracted by two reviewers using a standardized, pre-piloted data extraction form, and cross-checked for consistency. An artificial intelligence tool was employed as an adjunct reviewer to further verify the accuracy and completeness of the extracted data, following the AI-assisted data extraction workflow validated by Gartlehner et al. (22). Any discrepancies among the two reviewers and the AI output were resolved through discussion or consultation with a third reviewer. The following information was extracted from each included study: (1) first author and year of publication; (2) age of participants in both the intervention and control groups; (3) clinical stage of breast cancer; (4) cancer phase (during treatment or post-treatment); (5) intervention details, including intervention duration (weeks), frequency (sessions per week), session duration (minutes), supervision format, and type of mind-body exercise; (6) type of control condition; (7) sample size of the intervention and control groups; (8) outcome measures and corresponding assessment instruments. When outcome data were reported as medians and interquartile ranges, conversion to means and standard deviations was performed using the method recommended by Wan et al. (23). When data were presented only in graphical form, values were extracted using WebPlotDigitizer software. For studies with multiple follow-up time points, data from the immediate post-intervention assessment were used for the primary analysis.

### 2.5 Risk of bias assessment

The risk of bias of each included study was independently assessed by two reviewers using the Cochrane Risk of Bias 2.0 tool (RoB 2) (24), which evaluates five domains: (1) bias arising from the randomization process; (2) bias due to deviations from intended interventions; (3) bias due to missing outcome data; (4) bias in measurement of the outcome; (5) bias in selection of the reported result. Each domain was judged as “low risk of bias,” “some concerns,” or “high risk of bias,” and an overall risk of bias judgment was assigned to each study. Any disagreements were resolved through discussion or consultation with a third reviewer. The results were visualized using the robvis tool (25).

The overall quality of evidence for each outcome was evaluated using the Grading of Recommendations Assessment, Development and Evaluation (GRADE) framework (26). The GRADE assessment considered five downgrading factors: risk of bias, inconsistency, indirectness, imprecision, and publication bias. The certainty of evidence was classified into four levels: high, moderate, low, and very low.

### 2.6 Statistical analysis

All statistical analyses were performed using R software (version 4.3.0). Pairwise meta-analyses were conducted using the meta package. Bayesian network meta-analyses were performed using the gemtc package, which interfaces with JAGS (Just Another Gibbs Sampler, version 4.3.1). A two-sided *P* value < 0.05 was considered statistically significant for all analyses.

#### 2.6.1 Pairwise meta-analysis

Traditional pairwise meta-analyses were performed for all direct comparisons with available evidence using a frequentist framework. A random-effects model based on the restricted maximum likelihood (REML) estimation method was employed to account for between-study heterogeneity (27). The effect size was expressed as Hedges' g with 95% confidence intervals (CIs). For anxiety and depression outcomes, negative values indicated a favorable effect of the intervention (i.e., greater symptom reduction). For quality of life outcomes, positive values indicated a favorable effect (i.e., greater improvement). Statistical heterogeneity was assessed using Cochran's Q test (significance level set at *P* < 0.10) and the I^2^ statistic, with I^2^ values of 25%, 50%, and 75% representing low, moderate, and substantial heterogeneity, respectively (28). Depending on data availability, the following pre-specified subgroup analyses were planned to explore potential sources of heterogeneity: (1) clinical stage; (2) intervention duration (< 8 weeks vs. 8–12 weeks vs. >12 weeks); (3) exercise frequency, categorized by weekly volume as high (>150 min/week), moderate (90–150 min/week), and low (< 90 min/week); (4) total exercise dose, calculated as the product of intervention duration and weekly volume, categorized as high, moderate, and low.

#### 2.6.2 Network meta-analysis

Bayesian network meta-analyses were conducted to simultaneously compare all mind-body exercise modalities and control conditions (29). Both fixed-effects and random-effects models were fitted for each outcome, with effect sizes expressed as standardized mean differences (SMDs) and 95% credible intervals (CrIs). Because different instruments were used to assess quality of life, anxiety, and depression across studies, SMDs were used to standardize effects across scales. Outcome directions were harmonized before analysis, with positive values indicating greater improvement in quality of life and negative values indicating greater reductions in anxiety and depression. Non-informative prior distributions were specified: *N*(0, 10,000) for treatment effects and Uniform(0, 2) for the between-study heterogeneity standard deviation in the random-effects model. For each model, three Markov chain Monte Carlo chains were run with 100,000 iterations after a burn-in of 50,000 iterations (thinning interval = 10). Convergence was assessed via trace plots, density plots, and the Brooks-Gelman-Rubin diagnostic (potential scale reduction factor ≈ 1.0) (30).

Model selection was based on comparison of the deviance information criterion (DIC), residual deviance (Dres), and effective number of parameters (pD), supplemented by leverage plot inspection; a DIC difference exceeding 5 was considered meaningful, and adequate model fit was indicated by a Dres approximately equal to the total number of data points (31). The transitivity assumption was assessed qualitatively by comparing the distribution of potential effect modifiers across intervention nodes, including clinical stage, treatment phase, participant age, intervention duration, exercise frequency, total exercise dose, supervision format, control condition, and outcome assessment instruments. The consistency assumption was evaluated globally by comparing the DIC of consistency and inconsistency models (difference < 5 supporting consistency) and locally using the node-splitting method (32).

Network geometry was visualized using network plots, with node size proportional to total sample size and edge thickness proportional to the number of studies. A league table was constructed to present all pairwise SMDs with 95% CrIs. Ranking probabilities were summarized using Surface Under the Cumulative Ranking Curve (SUCRA) values, where higher values indicated a greater probability of achieving a higher rank. SUCRA values were interpreted as probabilistic rankings rather than as evidence of definitive clinical superiority, particularly when differences between active interventions were not statistically significant (33). Publication bias was assessed by visual inspection of comparison-adjusted funnel plots (34).

## 3 Results

### 3.1 Study selection and characteristics

**Figure 1:**
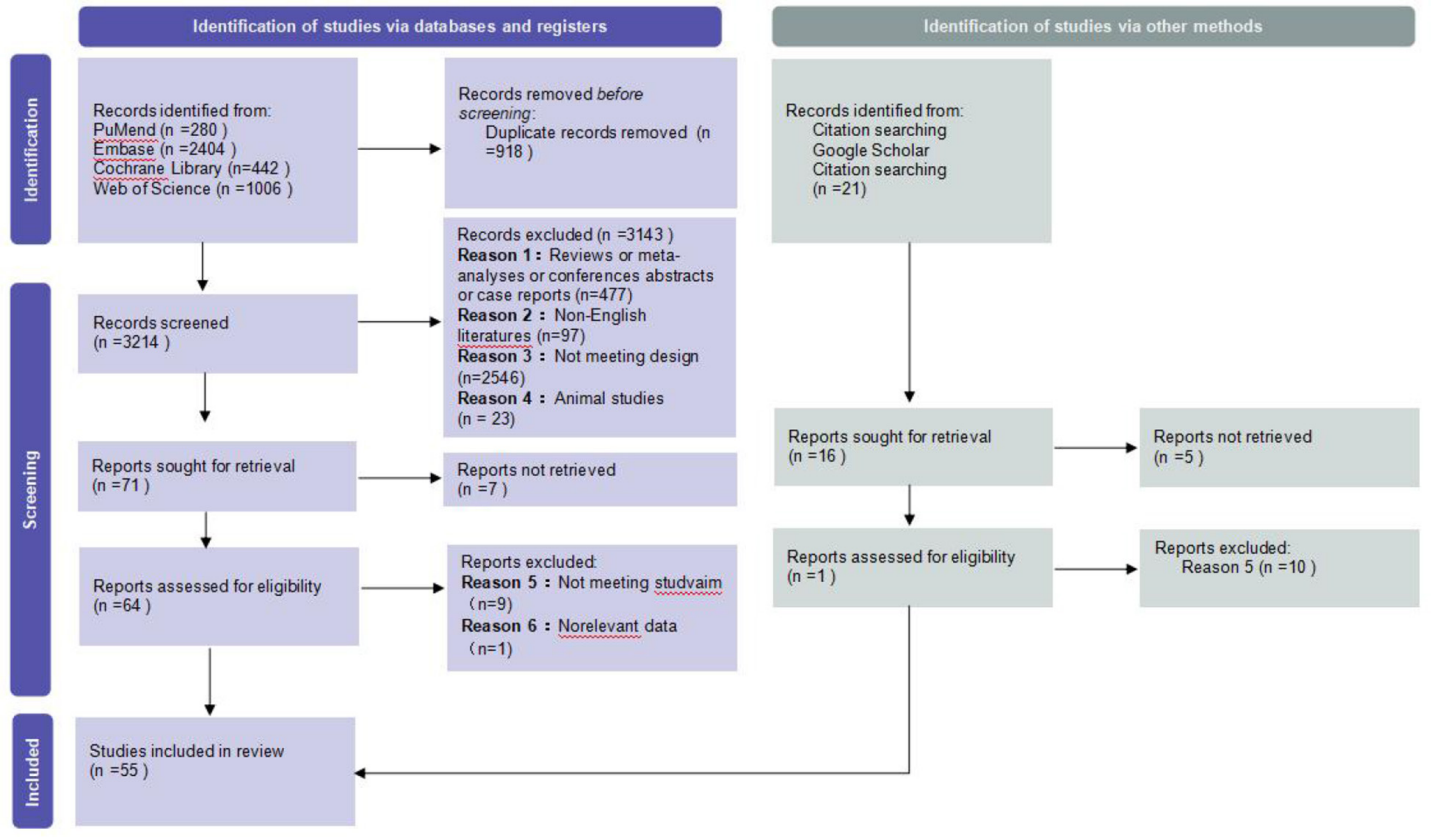
PRISMA flow diagram of the study selection process.

The systematic search yielded 4,132 records from four databases (PubMed 280, Embase 2,404, Cochrane Library 442, Web of Science 1,006) and 21 from other sources. After removing 918 duplicates, 3,214 records were screened, of which 3,143 were excluded. Of 75 full-text articles assessed, 20 were excluded, and 55 RCTs (54 from databases, 1 from other sources) were included in the final analysis (Figure 1).

The 55 trials encompassed 3,770 participants (1,833 interventions, 1,937 control) (Supplementary Table S2). Most studies enrolled patients with stage 0–III breast cancer. The interventions comprised Yoga (45.5%), Dance (18.2%), Tai Chi (12.7%), Pilates (12.7%), Qigong (7.3%), and two three-arm trials (3.6%) incorporating both Pilates and Dance. Intervention durations ranged from 3 to 24 weeks, with 12 weeks being the most common (*n* = 18). The most frequently used outcome measures were the FACT-B and EORTC QLQ-C30 for quality of life, the HADS for anxiety, and the BDI and CES-D for depression.

### 3.2 Risk of bias assessment

**Figure 2:**
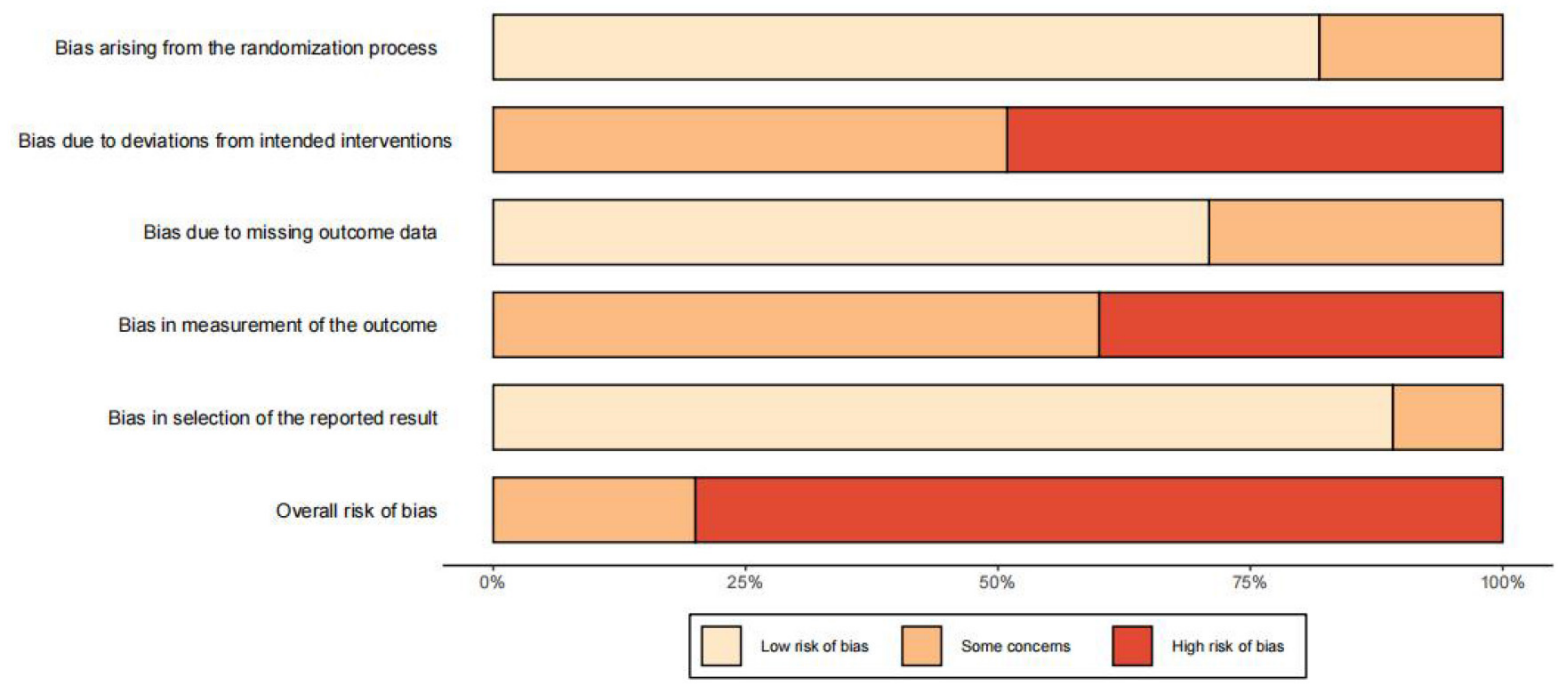
Risk of bias summary of included studies.

The risk of bias assessment is summarized in Figure 2, with detailed judgments for each study provided in Supplementary Table S3. Most studies were rated as low risk for the randomization process (D1, 80%) and selection of reported results (D5, 75%). The primary sources of bias were related to blinding: deviations from intended interventions (D2, 45% high risk) due to the infeasibility of blinding participants to exercise allocation, and measurement of outcomes (D4, 30% high risk) due to reliance on self-reported questionnaires in unblinded trials. Overall, 55% of studies were judged as high risk of bias, predominantly driven by the inherent blinding limitations of exercise intervention trials.

### 3.3 Pairwise meta-analysis results

**Figure 3:**
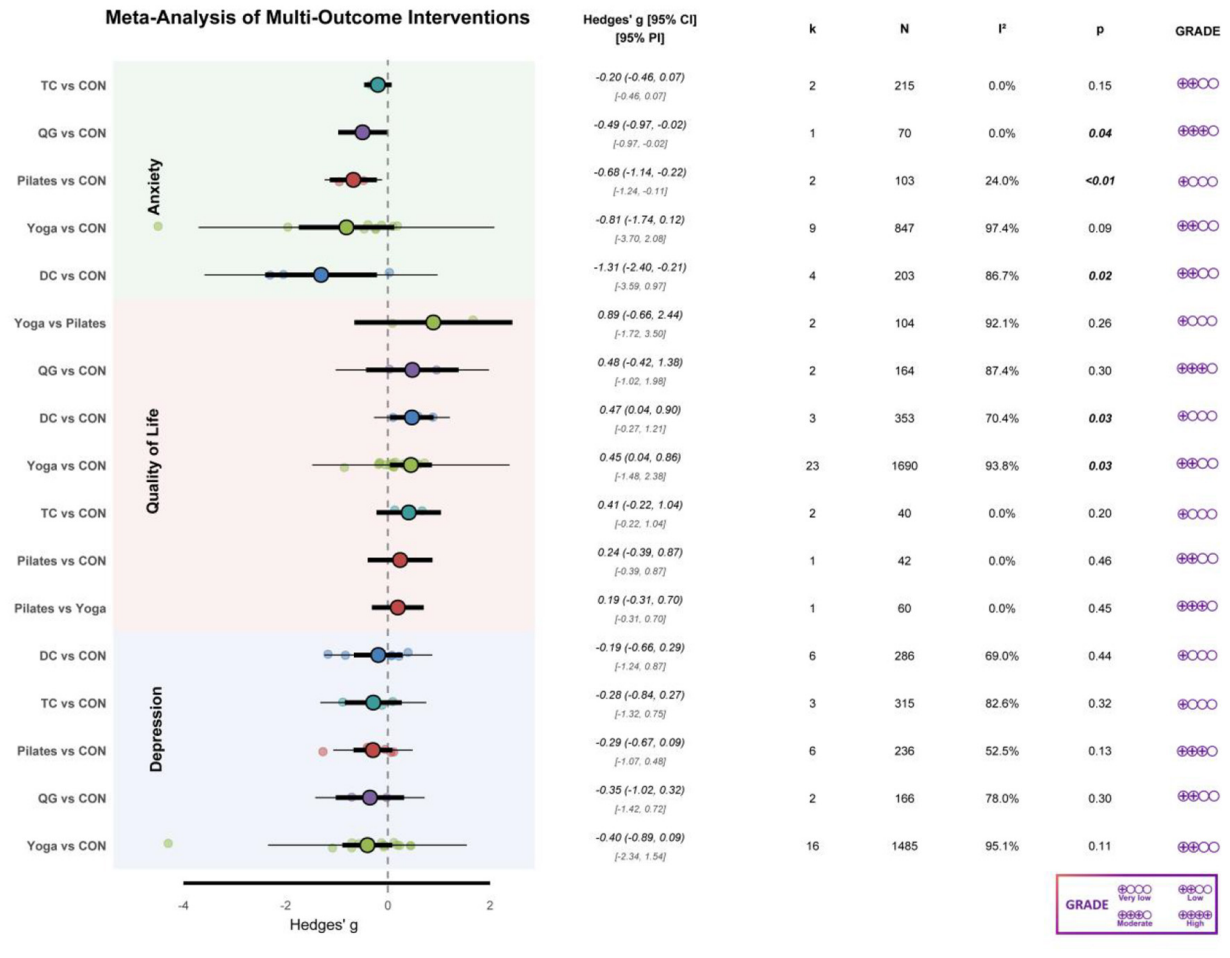
Pairwise meta-analysis of mind-body exercises vs. control for anxiety, quality of life, and depression. Effect sizes are expressed as Hedges' g with 95% confidence intervals (CI) and 95% prediction intervals (PI). The dashed line represents the null effect. GRADE evidence certainty is rated as high, moderate, low, or very low. k, number of studies; *N*, total participants; CON, control; DC, Dance; TC, Tai Chi; QG, Qigong.

The results of pairwise meta-analyses are presented in Figure 3. For anxiety, Dance (g = −1.31, 95% CI [−2.40, −0.21], p = 0.02), Pilates (g = −0.68, 95% CI [−1.14, −0.22], *p* < 0.01), and Qigong (g = −0.49, 95% CI [−0.97, −0.02], *p* = 0.04) significantly reduced symptoms compared with CON, whereas Yoga (*p* = 0.09) and Tai Chi (*p* = 0.15) did not; substantial heterogeneity was observed for Yoga and Dance, with low heterogeneity for the remaining comparisons. For quality of life, Yoga (g = 0.45, 95% CI [0.04, 0.86], *p* = 0.03) and Dance (g = 0.47, 95% CI [0.04, 0.90], *p* = 0.03) significantly improved outcomes, while Qigong (*p* = 0.30), Tai Chi (*p* = 0.20), and Pilates (*p* = 0.46) did not; substantial heterogeneity was present for Yoga and Qigong, with moderate heterogeneity for Dance. For depression, no intervention reached statistical significance (all *p* > 0.05); moderate-to-substantial heterogeneity was observed across all comparisons. GRADE evidence certainty was moderate for Dance on quality of life and Pilates on depression; the remaining comparisons were rated as very low, primarily downgraded due to risk of bias, inconsistency, and imprecision.

**Figure 4:**
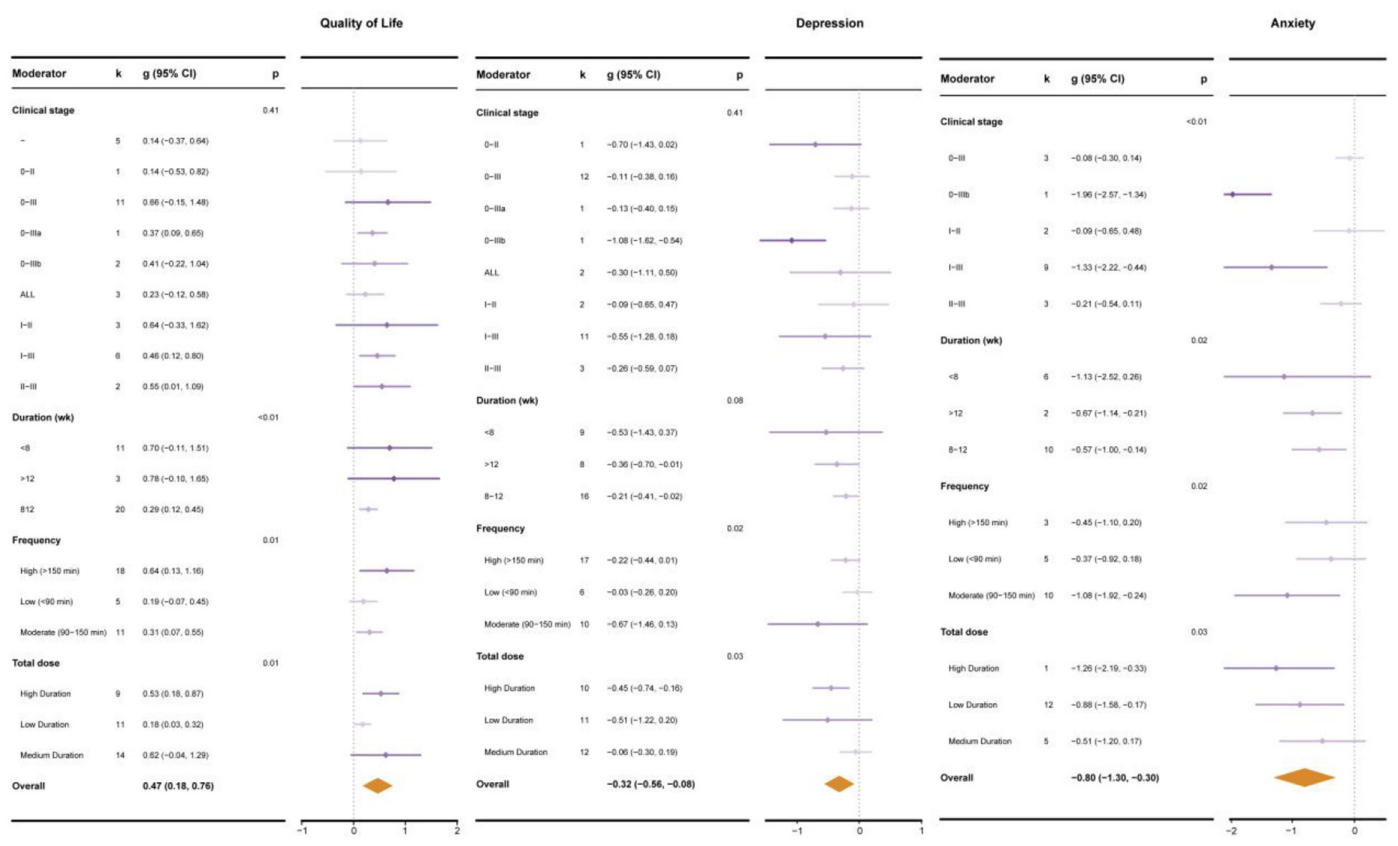
Subgroup analyses of pairwise meta-analyses. Effect sizes are expressed as Hedges' g with 95% confidence intervals. The diamond represents the overall pooled effect. *p*-values for subgroup differences are shown to the right of each moderator. k = number of studies.

Subgroup analyses were conducted by clinical stage, duration, frequency, and total dose (Figure 4). For quality of life, significant moderating effects were identified for duration (*p* < 0.01), frequency (*p* = 0.01), and total dose (*p* = 0.01), with high-frequency (>150 min/week; g = 0.64, 95% CI [0.13, 1.16]) and high-dose (g = 0.53, 95% CI [0.18, 0.87]) interventions yielding the largest effects; clinical stage was not significant (*p* = 0.41). For depression, frequency (*p* = 0.02) and total dose (*p* = 0.03) were significant moderators, with high-dose interventions showing the largest effect (g = −0.45, 95% CI [−0.74, −0.16]). For anxiety, all four moderators were significant: clinical stage (*p* < 0.01), duration (*p* = 0.02), frequency (*p* = 0.02), and total dose (*p* = 0.03), with stage I–III (g = −1.33, 95% CI [−2.22, −0.44]) and moderate-frequency interventions (g = −1.08, 95% CI [−1.92, −0.24]) showing the greatest reductions. Given the observational nature of subgroup analyses and the limited number of studies within some subgroups, these findings should be interpreted with caution as exploratory rather than confirmatory.

### 3.4 Network meta-analysis results

Both fixed-effects and random-effects models were fitted for each outcome. DIC comparison favored the random-effects model for all three outcomes, with DIC differences exceeding 5 in each case (Supplementary File 4). Convergence was confirmed by trace plots and density plots showing adequate mixing across three chains. Qualitative assessment of transitivity suggested that the included studies were generally comparable in terms of population and outcome domains; however, some imbalance existed across intervention nodes in clinical characteristics, intervention dose, outcome instruments, and the number of available studies. Global consistency was supported by comparing consistency and inconsistency models, with DIC differences less than 5 for all three outcomes; node-splitting analyses revealed no significant local inconsistency for any comparison (all *p* > 0.05).

**Figure 5:**
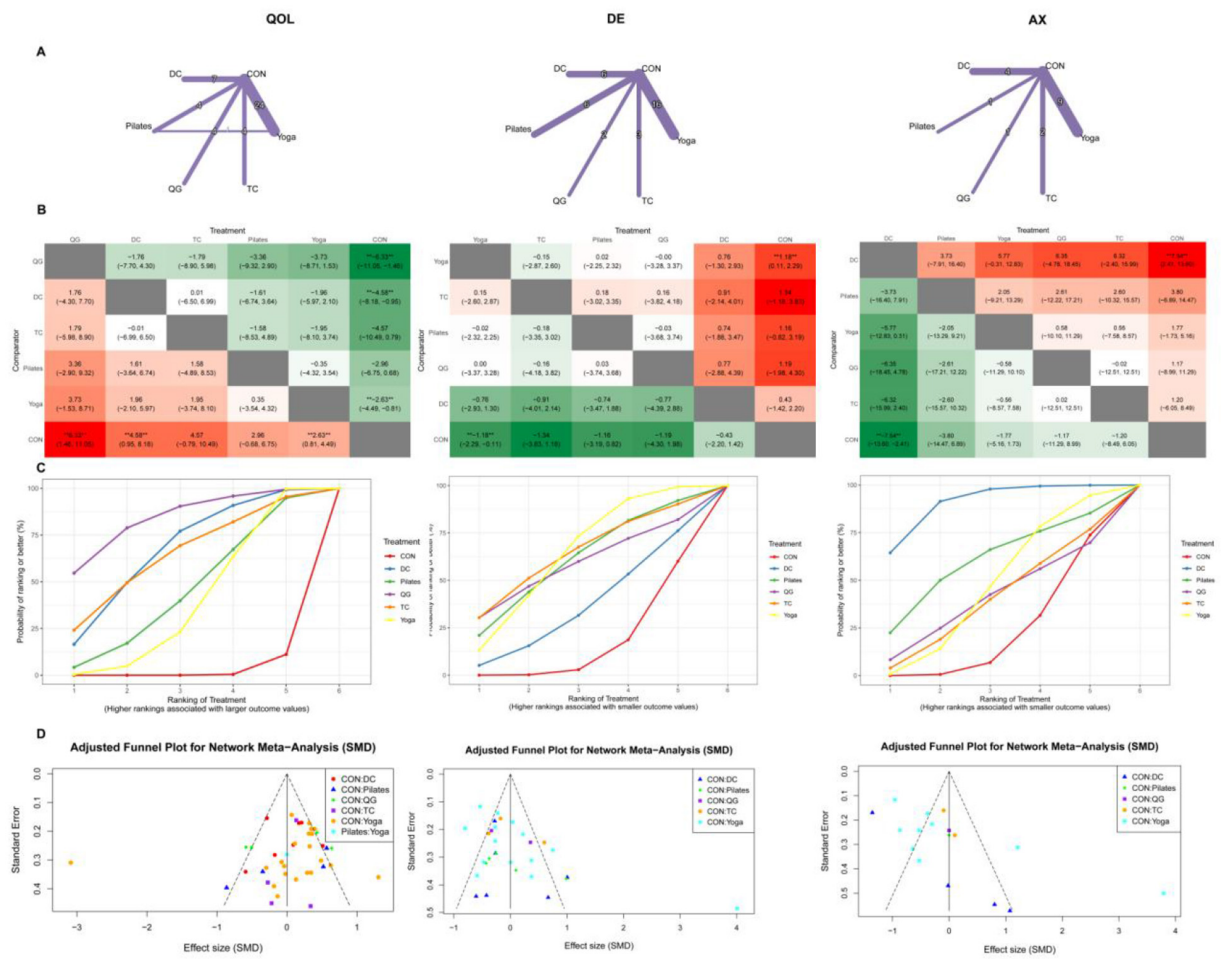
Network meta-analysis results. **(A)** Network plots; **(B)** League tables; **(C)** SUCRA rankograms; **(D)** Comparison-adjusted funnel plots. Node size reflects the number of studies; edge thickness represents direct comparisons. Statistically significant comparisons are marked with asterisks (**p < 0.05). QoL, quality of life; DE, depression; AX, anxiety; CON, control; DC, Dance; TC, Tai Chi; QG, Qigong.

The network geometry for quality of life, depression, and anxiety is shown in Figure 5A. For quality of life, Qigong (SMD = 6.33, 95% CrI [1.46, 11.05], *p* < 0.05), Dance (SMD = 4.58, 95% CrI [0.95, 8.18], *p* < 0.05), and Yoga (SMD = 2.63, 95% CrI [0.81, 4.49], *p* < 0.05) favored the intervention over control; however, no significant differences were detected between active interventions (Figure 5B). In the ranking probability analysis, Qigong showed the highest ranking probability (83.9%), followed by Dance (66.7%), Tai Chi (64.1%), Pilates (44.7%), and Yoga (38.4%) (Figure 5C). Given the wide credible interval and limited direct evidence for some nodes, particularly Qigong, this ranking should be interpreted cautiously.

For depression, only Yoga favored the intervention over control (SMD = −1.18, 95% CrI [−2.29, −0.11], *p* < 0.05); The SUCRA results suggested similar ranking probabilities for Yoga (64.3%) and Tai Chi (64.1%), followed by Pilates (60.7%), Qigong (58.3%), and Dance (36.4%). Therefore, these rankings should not be interpreted as evidence that one active modality was definitively superior to another.

For anxiety, only Dance favored the intervention over control (SMD = −7.54, 95% CrI [−13.60, −2.41], *p* < 0.05); no other comparison reached statistical significance. In the ranking probability analysis, Dance showed the highest ranking probability (90.6%), followed by Pilates (59.9%), Yoga (46.9%), Qigong (40.3%), and Tai Chi (39.7%). However, the large effect estimate and wide credible interval indicate that this finding should be interpreted cautiously, as it may be influenced by limited evidence, between-study heterogeneity, and differences in measurement instruments.

Visual inspection of the comparison-adjusted funnel plots (Figure 5D) did not reveal substantial asymmetry, suggesting no major publication bias. Taken together, the network meta-analysis suggested that several mind-body exercise modalities may be more effective than control conditions for specific outcomes. Nevertheless, because no statistically significant differences were observed between active interventions, and because some estimates were large with wide credible intervals, the SUCRA rankings should be regarded as exploratory ranking probabilities rather than evidence of definitive clinical superiority.

## 4 Discussion

This study integrated both pairwise and Bayesian network meta-analysis to compare the potential effects of five mind-body exercises—Yoga, Tai Chi, Qigong, Pilates, and Dance—on quality of life, anxiety, and depression in breast cancer patients. A total of 55 RCTs involving 3,770 participants were included. The pairwise meta-analysis suggested that Dance, Pilates, and Qigong were associated with greater reductions in anxiety, while Yoga and Dance were associated with greater improvements in quality of life compared with control; however, no intervention reached statistical significance for depression. The NMA results suggested that Qigong, Dance, and Yoga favored quality of life compared with control; Dance favored anxiety reduction compared with control; and Yoga favored depression reduction compared with control. However, no statistically significant differences were observed between any pair of active interventions across all three outcomes. Therefore, the SUCRA results should be interpreted as exploratory ranking probabilities rather than evidence of definitive superiority among active modalities. This cautious interpretation is further supported by the low to very low certainty of evidence, the high proportion of studies at high risk of bias, and the predominantly star-shaped network with limited head-to-head comparisons. Subgroup analyses suggested that exercise frequency and total dose may be relevant moderators across outcomes, with higher-dose interventions tending to show larger effects.

Our findings are broadly consistent with the existing literature, but they should be interpreted cautiously. The umbrella review by Wen et al. (13) reported evidence that Yoga may reduce depressive symptoms among breast cancer patients, which is generally consistent with our finding that Yoga favored depression reduction compared with control. Pi et al. (18) conducted a network meta-analysis and found that Baduanjin, a form of Qigong, showed a relatively large effect size for improving quality of life, which is compatible with the relatively high ranking probability of Qigong in our analysis. However, this does not establish Qigong as definitively superior to other active mind-body exercise modalities. A recent umbrella review by Xu et al. (35) also suggested that Qigong and Tai Chi may improve cancer patients' overall quality of life, physiological functioning, and psychological wellbeing. For anxiety, Wang et al. (19) reported that mind-body exercise had a high ranking probability among exercise types. This is broadly consistent with our finding that Dance—a modality emphasizing rhythmic movement, social interaction, and emotional expression—showed the highest ranking probability for anxiety reduction. Nevertheless, given the large effect estimate and wide credible interval, this result should be regarded as an exploratory signal rather than conclusive evidence of superiority. Han et al. (36) similarly reported that mind-body and combined exercises may improve quality of life in breast cancer patients, whereas resistance exercise alone showed less consistent evidence. Zhang et al. (37) also reported that exercise interventions were associated with reductions in depression and anxiety among breast cancer survivors, with mind-body exercise showing potential benefits. Additionally, a large-scale network meta-analysis by Wu et al. (38) evaluating the impact of different exercise types across cancer populations found that mind-body exercise was associated with reduced depression, and Yoga showed a high ranking probability for alleviating depressive symptoms. These findings are consistent with the direction of our results, but they should not be interpreted as confirming definitive superiority of Yoga over other active modalities. The dose-response pattern observed in our subgroup analyses is also consistent with the individual participant data meta-analysis by Kenkhuis et al. (39), which reported that the effects of exercise on depression and anxiety may vary according to patient, clinical, and intervention characteristics in cancer survivors. Compared with these prior NMAs, our analysis is distinguished by the simultaneous evaluation of three psychological outcomes within a single analytic framework, the explicit isolation of five MBE modalities, and the integration of intervention-dose moderator analyses alongside the network estimates.

The included modalities were grouped as mind-body exercises because they share several core features, including low-to-moderate physical movement, attentional focus, breath regulation or body awareness, and integration of physical and psychological processes (10, 11). At the same time, these modalities are conceptually heterogeneous: Yoga emphasizes postures, controlled breathing, and meditative awareness; Tai Chi and Qigong emphasize slow coordinated movement, breath regulation, and mindful attention; Pilates focuses more on controlled core-based movement and body alignment; and Dance additionally involves rhythm, music, emotional expression, and social interaction (10, 40, 41). Several potential mechanisms may help explain why mind-body exercises could influence psychological outcomes, although these mechanisms should not be interpreted as evidence of definitive superiority among modalities. First, mind-body exercises may modulate the hypothalamic-pituitary-adrenal (HPA) axis, thereby reducing cortisol secretion and attenuating stress-induced physiological arousal (42). A systematic review and network meta-analysis by Li et al. (43) reported that Yoga was associated with the largest reduction in cortisol levels among exercise modalities in individuals with psychological distress, which may provide a plausible biological explanation for the relatively favorable ranking of Yoga for depression in our analysis. Yoga's integration of sustained postures, controlled breathing, and meditative awareness may enhance parasympathetic nervous system activity, decrease sympathetic tone, and restore autonomic balance, which may be relevant to improvements in depressive symptoms (44). Second, mind-body interventions have been shown to reduce levels of pro-inflammatory cytokines (e.g., IL-6, TNF-α, and CRP) and increase anti-inflammatory and neurotrophic factors (e.g., BDNF), suggesting a possible immunomodulatory pathway linking exercise to psychological improvement (45). A scoping review further indicated that yoga-based interventions were associated with increased serum BDNF and reduced cortisol and IL-6 levels in patients with depression, providing preliminary biological evidence for potential antidepressant mechanisms of yoga-based mind-body exercises (46). Third, the relatively high ranking probability of Dance for anxiety reduction may be attributable to its combination of rhythmic movement, musical stimulation, social engagement, and creative expression, which may promote emotional release and reduce psychological tension beyond the effects of movement alone (40). Finally, the meditative and breath-regulation components inherent in Qigong and Tai Chi may enhance mindfulness and interoceptive awareness, thereby potentially contributing to improved quality of life through better self-regulation and emotional resilience (41). Beyond these mechanistic effects, mind-body exercises are also accessible (minimal equipment), scalable (group or digital delivery), and well suited for long-term self-management in breast cancer survivorship care. Nevertheless, these mechanistic explanations remain hypothetical and should be confirmed in future mechanistic and head-to-head comparative trials.

This study has several limitations. First, the certainty of evidence was low to very low for most comparisons, and 55% of included studies were judged to be at high risk of bias. This was mainly related to the difficulty of blinding participants and personnel in exercise trials and the frequent use of self-reported outcomes. Therefore, the observed effects and ranking probabilities should be interpreted cautiously. Second, substantial heterogeneity was observed across several comparisons. This heterogeneity may be related to differences in participant characteristics, cancer stage, treatment phase, intervention duration, exercise frequency, total exercise dose, supervision format, and control conditions. Third, quality of life, anxiety, and depression were assessed using different instruments across studies. Although SMDs were used to standardize effects across scales, variability in measurement tools may still have affected the comparability and interpretation of pooled estimates. Fourth, some intervention nodes were supported by a limited number of studies, such as Qigong, and the network was predominantly star-shaped, with few direct head-to-head comparisons between active modalities. As a result, some NMA estimates, especially large estimates with wide credible intervals, may be unstable and influenced by indirect evidence, small-study effects, or residual heterogeneity. Fifth, although statistical inconsistency was not detected, the transitivity assumption could not be fully verified because potential effect modifiers were not evenly distributed across all intervention nodes. Finally, the review was limited to English-language publications. This restriction may be particularly relevant for interventions such as Qigong and Tai Chi, which originated and are widely practiced in Chinese-speaking contexts. Excluding non-English studies may have affected the comprehensiveness of the evidence base, the distribution of studies across intervention nodes, and the resulting ranking probabilities.

## 5 Conclusion

This systematic review with pairwise and network meta-analysis of 55 RCTs suggests that mind-body exercises may improve quality of life, anxiety, and depression in patients with breast cancer. Compared with control conditions, Qigong, Dance, and Yoga favored improvements in quality of life, Dance favored anxiety reduction, and Yoga favored depression reduction. However, no statistically significant differences were observed between active interventions, and SUCRA rankings should therefore be interpreted as exploratory ranking probabilities rather than evidence of definitive superiority. Higher exercise frequency and total dose may be associated with greater improvements, although these findings should be interpreted cautiously. Clinicians may consider mind-body exercises as adjunctive supportive care options, but modality-specific recommendations should be made cautiously given the low to very low certainty of evidence and the high risk of bias in many included trials. Future high-quality, adequately powered head-to-head RCTs are needed to confirm the comparative effects of specific mind-body exercise modalities.

## Data Availability

The data analyzed in this study is subject to the following licenses/restrictions: The datasets generated during and/or analyzed during the current study are available from the corresponding author on reasonable request. Requests to access these datasets should be directed to TZ, zhoutian912@scu.edu.cn.
