# Supplementary material for: Comparative efficacy of mind-body exercises on quality of life, anxiety, and depression in patients with breast cancer: a systematic review with pairwise and network meta-analysis of randomized controlled trials
Source: Front Public Health. 2026 Jul 16;14:1830453. doi: 10.3389/fpubh.2026.1830453 (PMC13421397; doi:10.3389/fpubh.2026.1830453)
Supplement: Supplementary file 1 [file Data_Sheet_1.docx]

[Supplementary File S1 Search Strategy 2](#_Toc12095)

[Supplementary File S1 Search Strategy Characteristics of Included Studies 4](#_Toc31850)

[Supplementary File S3 Risk of bias assessment 13](#_Toc26357)

[Supplementary File S4 Network Meta-Analysis 17](#_Toc11491)

# **Supplementary File 1 Search Strategy**

## Table S1. Supplementary Material: Search Strategy

| **Category** | **Search Keywords (OR)** |
| --- | --- |
| **P (Population: Cancer)** | Cancer OR Oncology OR Neoplasm OR Carcinoma OR Malignancy OR Tumor OR Tumour OR Breast Cancer OR Lung Cancer OR Prostate Cancer OR Colorectal Cancer OR Hematologic Neoplasms OR Leukemia OR Lymphoma |
| **I (Intervention: Mind-Body Exercise)** | mind-body exercise OR mind body exercise OR mind-body practice OR Yoga OR Vinyasa OR Tai ji OR Tai-ji OR Tai Chi OR Chi, Tai OR Tai Ji Quan OR Taijiquan OR Qigong OR Qi Gong OR Baduanjin OR Wuqinxi OR Pilates OR Dance OR Dance therapy OR Yijinjing OR Liuzijue |
| **O (Outcomes: Psychological & QoL)** | Anxiety OR Depression OR quality of life OR Angst OR Nervousness OR Hypervigilance OR Anxieties, Social OR Anxiety, Social OR Anxiousness OR QOL OR QoL OR HRQoL OR HRQOL OR life quality OR PDQ-39 OR Parkinson's Disease Questionnaire OR PDQ-8 OR SF-36 OR Short Form 36 OR health survey OR EQ-5D OR EuroQol OR WHO-QOL OR World Health Organization Quality of Life |
| **S (Study Design: RCT)** | Randomized Controlled Trials as Topic OR Randomized Controlled Trial OR random* OR clinic* OR trial* |

# **Supplementary File 2 Search Strategy Characteristics of Included Studies**

## Table S2. Characteristics of Included Studies

| **NO** | **study** | **year** | **Age**  **（T/C）** | **Age**  **（T/C）** | **Clinical stage** | **Cancer phase** | **week** | **perweek** | **Duration**  **(min)** | **Week Min** | **Total Min** | **Dose** | **Supervision** | **Intervention** | **Control** | **n (intervention)** | **n (control)** | **Outcome** | **Assessment Tool** |
| --- | --- | --- | --- | --- | --- | --- | --- | --- | --- | --- | --- | --- | --- | --- | --- | --- | --- | --- | --- |
| 1 | Sandel | 2005 | 59.7±9.8 | 59.5±13.3 | II-III | Post-treatment | 12 | 1.5 | 67.5 | 101.25 | 1215 | 354.375 | 1 | Dance | CON | 19 | 19 | 1 | FACT-B |
| 3 | Banerjee | 2007 | 47±1.1 | 43±1.5 | I-III | Post-treatment or During treatment | 6 | 1 | 90 | 90 | 540 | 225 | 1 | Yoga | CON | 35 | 23 | 2,3 | HADS-A、HADS-D |
| 5 | Mustian | 2008 | 52±9 | 52±9 | 0-IIIb | Post-treatment or Survivorship | 12 | 3 | 60 | 180 | 2160 | 540 | 1 | TC | CON | 11 | 10 | 1 | FACT-F |
| 6 | Rao | 2008 | 30-70 | 30-70 | II-III | Pretreatment | 4 | 1 | 60 | 60 | 240 | 150 | 1 | Yoga | CON | 33 | 36 | 1，2，3 | STAI、BDI、FLIC |
| 7 | Danhauer | 2009 | 54.3±9.6 | 57.2±10.2 | ALL | During treatment or Post-treatment | 10 | 1 | 75 | 75 | 750 | 187.5 | 1 | Yoga | CON | 22 | 22 | 1，2 | FACT-B, SF-12、CES-D |
| 8 | Raghavendra | 2009 | 46±9.13 | 48.45±10.21 | II-III | During treatment | 6 | 3 | 60 | 180 | 1080 | 450 | 1 | Yoga | CON | 44 | 44 | 2,3 | HADS |
| 9 | Chandwani | 2010 | 51.39±7.97 | 40.2±9.96 | 0-IIIb | During treatment | 6 | 2 | 60 | 120 | 720 | 300 | 1 | Yoga | CON | 30 | 31 | 2,3 | CES-D、STAI |
| 10 | Eyigor | 2010 | 48.52±7.62 | 49.73±8.71 | ALL | Post-treatment or Survivorship | 8 | 3 | 60 | 180 | 1440 | 504 | 1 | Pilates | CON | 27 | 15 | 1，2 | EORTC QLQ-C30、BDI |
| 12 | Haddad | 2011 | 52.38±1.35 | 52.11±1.34 | 0-III | During treatment | 6 | 3 | 60 | 180 | 1080 | 450 | 1 | Yoga | CON | 53 | 54 | 1，2 | SF-36，CES-D |
| 14 | Bower | 2012 | 54.4±5.7 | 53.3±4.9 | 0-II | Post-treatment or Survivorship | 12 | 2 | 90 | 180 | 2160 | 450 | 1 | Yoga | CON | 16 | 15 | 2 | BDI-II |
| 15 | Littman | 2012 | 33-74 | 33-74 | 0-III | Post-treatment | 24 | 5 | 75 | 375 | 9000 | 937.5 | 1 | Yoga | CON | 32 | 31 | 1 | FACT-G |
| 17 | Pruthi | 2012 | 56.08±26.99 | 60.54±22.08 | 0-III | During treatment or Post-treatment | 12 | 1 | 60 | 60 | 720 | 150 | 1 | Yoga | CON | 15 | 15 | 1，2，3 | FACT-B，POMS-SF |
| 18 | Sprod | 2012 | 54.33±3.55 | 52.70±2.11 | 0-IIIb | Post-treatment or Survivorship | 12 | 3 | 60 | 180 | 2160 | 540 | 1 | TC | CON | 9 | 10 | 1 | HRQOL |
| 19 | Chen | 2013 | 45.3±6.3 | 44.7±9.7 | 0-III | During treatment | 6 | 5 | 40 | 200 | 1200 | 600 | 1 | QG | CON | 49 | 47 | 1，2 | FACT-G，CES-D |
| 20 | Siedentopf | 2013 | 37-84 | 37-84 | 0-III | Post-treatment | 5 | 2 | 75 | 150 | 750 | 375 | 1 | Yoga | CON | 49 | 44 | 1 | EORTC QLQ-BR23 |
| 21 | Andysz | 2014 | 54.8±7.4 | 58.6±10.8 | I-III | Post-treatment or Survivorship | 10 | 1 | 90 | 90 | 900 | 225 | 1 | Yoga | CON | 12 | 16 | 1 | QLQ-C30 |
| 24 | Kiecolt | 2014 | 51.8±9.8 | 51.3±8.7 | 0-IIIa | Post-treatment or Survivorship | 12 | 2 | 90 | 180 | 2160 | 450 | 1 | Yoga | CON | 100 | 100 | 1，2 | SF-36，CES-D |
| 25 | Loudon | 2014 | 55.1±2.5 | 60.5±3.6 | - | Survivorship | 8 | 1 | 90 | 90 | 720 | 225 | 1 | Yoga | CON | 12 | 11 | 1 | QOL |
| 26 | Ratcliff | 2014 | 52.38±1.35 | 52.11±1.34 | 0-III | During treatment | 6 | 3 | 60 | 180 | 1080 | 450 | 1 | Yoga | CON | 53 | 54 | 1，2 | SF-36，CES-D |
| 27 | Cramer | 2015 | 49.2±4.8 | 50.0±6.7 | I-III | Post-treatment or Survivorship | 12 | 1 | 90 | 90 | 1080 | 225 | 1 | Yoga | CON | 20 | 20 | 1，2，3 | FACT-B,HADS |
| 28 | Danhauer | 2015 | 54.3±42.79 | 46.8±28.1 | I-III | Post-treatment | 10 | 1 | 75 | 75 | 750 | 187.5 | 1 | Yoga | CON | 22 | 18 | 1，2 | FACT-B，CES-D |
| 29 | Larkey | 2015 | 57.7±8.94 | 59.8±8.93 | 0-III | Post-treatment or Survivorship | 12 | 1.5 | 60 | 90 | 1080 | 270 | 1 | TC | CON | 42 | 45 | 2 | BDI |
| 30 | Rao | 2015 | 30-70 | 30-70 | II-III | During treatment | 24 | 3 | 60 | 180 | 4320 | 450 | 1 | Yoga | CON | 33 | 36 | 2 | BDI |
| 31 | Vardar | 2015 | 49.89±4.65 | 47.38±7.57 | - | Survivorship | 6 | 3 | 90 | 270 | 1620 | 675 | 1 | Yoga | CON | 19 | 21 | 1 | EORTC QOL-C30 |
| 33 | Ho | 2016 | 48.6±7.7 | 49.1±8.7 | 0-III | During treatment | 3 | 2 | 90 | 180 | 540 | 630 | 1 | Dance | CON | 69 | 70 | 1，2，3 | FACT-B,HADS |
| 34 | Lotzke | 2016 | 51.0±11.0 | 51.4±11.1 | I-III | During treatment | 12 | 3 | 60 | 180 | 2160 | 450 | 1 | Yoga | CON | 45 | 47 | 1 | EORTC QLQ-C30 |
| 35 | Ratcliff | 2016 | - | - | 0-III | During treatment | 6 | 3 | 60 | 180 | 1080 | 450 | 1 | Yoga | CON | 55 | 54 | 1，2 | SF-36，CES-D |
| 36 | Stan | 2016 | 61.4±7.0 | 63.0±9.3 | 0-II | Survivorship | 12 | 4 | 90 | 360 | 4320 | 900 | 1 | Yoga | CON | 18 | 16 | 1 | FACT-B |
| 37 | Odynets | 2019 | 59.10±1.37 | 59.40±1.24 | I-II | Post-treatment | 24 | 3 | 60 | 180 | 4320 | 450 | 1 | Yoga | Pilates | 30 | 40 | 1 | FACT-B |
| 40 | Bem | 2021 | 53.33±8.58 | 57.50±13.02 | 0-III | During treatment | 16 | 3 | 60 | 180 | 2880 | 504 | 1 | Pilates | CON | 18 | 16 | 2 | BDI |
| 41 | Eyigor | 2021 | 51.40±10.6 | 50.7±7.6 | - | Survivorship | 10 | 2 | 60 | 120 | 1200 | 300 | 1 | Yoga | CON | 15 | 16 | 1 | EORTC QLQ-C30 |
| 42 | Leite | 2021 | T1:55.94 ± 10.98 T2:55.94 ± 10.98 | 55.94 ± 10.98 | 0-III | During treatment | 16 | 3 | 60 | 180 | 2880 | T1:504 T2:630 | 1 | T1:Pilates T2:Dance | CON | T1:18 T2:18 | 16 | 2 | BDI |
| 48 | He | 2022 | 47.99±8.62 | 48.32±10 | I-III | Post-treatment | 16 | 5 | 30 | 150 | 2400 | 525 | 1 | Dance | CON | 88 | 88 | 1 | FACT-B |
| 49 | Liao | 2022 | 53.12±7.02 | 54.63±8.44 | I-III | Post-treatment | 12 | 2 | 90 | 180 | 2160 | 540 | 1 | QG | CON | 33 | 35 | 1 | EORTC QLQ-C30 |
| 50 | Liu | 2022 | ＞18 | ＞18 | I-II | Post-treatment | 8 | 1 | 90 | 90 | 720 | 225 | 1 | Yoga | CON | 68 | 68 | 1，2，3 | FACT-B、HADS |
| 52 | Wei | 2022 | 40-75 | 40-75 | I-III | During treatment | 12 | 5 | 30 | 150 | 1800 | 450 | 1 | QG | CON | 35 | 35 | 1，2，3 | FACT-B,HADS |
| 54 | Zhang | 2022 | 47.79±5.14 | 47.20±7.65 | I-III | Post-treatment | 8 | 2 | 60 | 120 | 960 | 360 | 1 | TC | CON | 30 | 29 | 3 | SAS |
| 55 | Alejandra | 2023 | 57.02±8.7 | 55.84±10.32 | I-III | Post-treatment | 8 | 3 | 52.5 | 157.5 | 1260 | 551.25 | 1 | Dance | CON | 31 | 33 | 1 | EORTC QLQ-C30 |
| 56 | Chang | 2023 | 51.91±10.51 | 52.77±8.53 | I-II | During treatment | 15 | 5 | 35 | 175 | 2625 | 525 | 1 | QG | CON | 30 | 30 | 1 | EORTC QLQ-C30 |
| 58 | Oei | 2023 | 61.3±9.9 | 61.6±11.7 | I-III | Survivorship | 6 | 1 | - | - | - | - | 1 | Dance | CON | 50 | 108 | 1 | EORTC QLQ-C30 |
| 59 | Schad | 2023 | 61.7±9.4 | 59.3±11.0 | I-III | Survivorship | 6 | 1 | 60 | 60 | 360 | 210 | 1 | Dance | CON | 24 | 28 | 1 | EORTC QLQ-C30 |
| 60 | Leite | 2024 | 55.29±10.93 | 55.29±10.93 | 0-III | Post-treatment | 16 | 3 | 60 | 180 | 2880 | 504 | 1 | Pilates | CON | 18 | 16 | 1 | EORTC QLQ-C30 & BR-23 |
| 64 | Barcelos | 2024 | 58.7±9.9 | 58.7±9.9 | I-III | Post-treatment | 12 | 2 | 60 | 120 | 1440 | 420 | 1 | Dance | CON | 10 | 11 | 1，2 | BDI |
| 66 | Bertoli | 2025 | 55.2±7.6 | 55.4±6.6 | 0-III | During treatment | 24 | 3 | 60 | 180 | 4320 | 504 | 1 | Pilates | CON | 21 | 19 | 1 | EORTC QLQ-C30 & BR23 |
| 67 | Silveira | 2025 | 57.7±9.0 | 51.5±10.8 | I-III | During treatment | 12 | 2 | 60 | 120 | 1440 | 420 | 1 | Dance | CON | 11 | 12 | 2,3 | BDI、BAI |
| 68 | Lam | 2025 | 51.2±9.1 | 51.7±9.1 | I-II | Post-treatment | 12 | 1 | 60 | 60 | 720 | 150 | 1 | Yoga | CON | 148 | 148 | 1，2 | HADS |
| 69 | Larkey | 2025 | 59.3±8.9 | 60.2±8.4 | 0-III | Post-treatment | 8 | 1 | 60 | 60 | 480 | 180 | 1 | TC | CON | 78 | 78 | 1，2，3 | POMS-SF、FACT-COG |
| 70 | Petzold | 2025 | 59±9.9 | 51±10.8 | I-III | Survivorship | 12 | 2 | 60 | 120 | 1440 | 420 | 1 | Dance | CON | 10 | 11 | 2,3 | BDI、BAI |
| 73 | Yang | 2025 | 52.8±11.6 | 53.8±8.9 | I–IV | During treatment or Post-treatment | 12 | 1 | 60 | 60 | 720 | 210 | 1 | Dance | CON | 33 | 33 | 1 | EORTC QLQ-C30 & BR23 |
| 74 | Bertoli | 2025 | 55.2±7.6 | 55.4±6.6 | I-III | Survivorship | 24 | 3 | 60 | 180 | 4320 | 504 | 1 | Pilates | CON | 21 | 22 | 2,3 | HADS |
| 75 | Hülya | 2017 | 55.2±7.6 | 55.4±6.6 | 0-III | Survivorship | 24 | 3 | 60 | 180 | 4320 | 504 | 1 | Pilates | CON | 30 | 30 | 1,3 | EORTC QLQ-BR23、SAA |
| 76 | Boing | 2023 | T1:54.3±10.4 T2:55.0±9.9 | 56.8±11.2 | I-III | Post-treatment | 16 | 3 | 60 | 180 | 2880 | T1:504 T2:630 | 1 | T1:Pilates T2:Dance | CON | T1:25 T2:25 | 24 | 2 | BDI |
| 77 | Boing | 2021 | 53.33±8.58 | 57.5±13.02 | 0-III | Post-treatment | 16 | 3 | 60 | 180 | 2880 | 504 | 1 | Pilates | CON | 18 | 16 | 2 | BDI |
| 78 | Thongteratham | 2015 | 54.8±8.24 | 51.73±8.94 | 0-III | Post-treatment | 12 | 2 | 60 | 120 | 1440 | 360 | 1 | TC | CON | 15 | 15 | 1 | FACT-B |
| 79 | Yao | 2022 | 45.3±8.5 | 48.6±7.8 | I-III | During treatment | 8 | 3 | 60 | 180 | 1440 | 540 | 1 | TC | CON | 36 | 36 | 2 | PHQ-9 |

# **Supplementary File 3 Risk of bias assessment**

## Table S3. Risk of bias assessment

| **No** | **Study** | **D1** | **D2** | **D3** | **D4** | **D5** | **Overall** |
| --- | --- | --- | --- | --- | --- | --- | --- |
|  | **Sandel** | Low Risk | High Risk | Some Concerns | High Risk | Low Risk | High Risk |
|  | **Banerjee** | Low Risk | Some Concerns | Low Risk | Some Concerns | Low Risk | Some Concerns |
|  | **Mustian** | Low Risk | Some Concerns | Low Risk | High Risk | Low Risk | High Risk |
|  | **Rao** | Some Concerns | High Risk | Low Risk | Some Concerns | Low Risk | High Risk |
|  | **Danhauer** | Low Risk | Some Concerns | Low Risk | High Risk | Low Risk | High Risk |
|  | **Raghavendra** | Some Concerns | High Risk | Some Concerns | High Risk | Low Risk | High Risk |
|  | **Chandwani** | Low Risk | Some Concerns | Low Risk | Some Concerns | Low Risk | Some Concerns |
|  | **Eyigor** | Low Risk | High Risk | Low Risk | Some Concerns | Some Concerns | High Risk |
|  | **Haddad** | Low Risk | Some Concerns | Some Concerns | High Risk | Low Risk | High Risk |
|  | **Bower** | Low Risk | Some Concerns | Low Risk | Some Concerns | Low Risk | Some Concerns |
|  | **Littman** | Low Risk | High Risk | Low Risk | Some Concerns | Low Risk | High Risk |
|  | **Pruthi** | Low Risk | Some Concerns | Some Concerns | High Risk | Low Risk | High Risk |
|  | **Sprod** | Some Concerns | High Risk | Low Risk | Some Concerns | Low Risk | High Risk |
|  | **Chen** | Low Risk | Some Concerns | Low Risk | Some Concerns | Low Risk | Some Concerns |
|  | **Siedentopf** | Some Concerns | High Risk | Some Concerns | High Risk | Low Risk | High Risk |
|  | **Andysz** | Some Concerns | High Risk | Some Concerns | Some Concerns | Some Concerns | High Risk |
|  | **Kiecolt** | Low Risk | Some Concerns | Low Risk | Some Concerns | Low Risk | Some Concerns |
|  | **Loudon** | Low Risk | High Risk | Some Concerns | Some Concerns | Low Risk | High Risk |
|  | **Ratcliff** | Low Risk | Some Concerns | Low Risk | High Risk | Low Risk | High Risk |
|  | **Cramer** | Low Risk | Some Concerns | Low Risk | Some Concerns | Low Risk | Some Concerns |
|  | **Danhauer** | Low Risk | High Risk | Low Risk | Some Concerns | Low Risk | High Risk |
|  | **Larkey** | Low Risk | Some Concerns | Low Risk | Some Concerns | Low Risk | Some Concerns |
|  | **Rao** | Some Concerns | High Risk | Low Risk | High Risk | Low Risk | High Risk |
|  | **Vardar** | Low Risk | High Risk | Low Risk | Some Concerns | Some Concerns | High Risk |
|  | **Ho** | Low Risk | Some Concerns | Some Concerns | High Risk | Low Risk | High Risk |
|  | **Lotzke** | Low Risk | High Risk | Low Risk | Some Concerns | Low Risk | High Risk |
|  | **Ratcliff** | Low Risk | Some Concerns | Low Risk | High Risk | Low Risk | High Risk |
|  | **Stan** | Low Risk | Some Concerns | Some Concerns | High Risk | Low Risk | High Risk |
|  | **Odynets** | Some Concerns | High Risk | Low Risk | Some Concerns | Some Concerns | High Risk |
|  | **Bem** | Low Risk | Some Concerns | Low Risk | High Risk | Low Risk | High Risk |
|  | **Eyigor** | Low Risk | High Risk | Low Risk | Some Concerns | Low Risk | High Risk |
|  | **Leite** | Low Risk | Some Concerns | Some Concerns | High Risk | Low Risk | High Risk |
|  | **He** | Low Risk | Some Concerns | Low Risk | Some Concerns | Low Risk | Some Concerns |
|  | **Liao** | Low Risk | High Risk | Low Risk | Some Concerns | Low Risk | High Risk |
|  | **Liu** | Low Risk | Some Concerns | Low Risk | High Risk | Low Risk | High Risk |
|  | **Wei** | Low Risk | High Risk | Low Risk | Some Concerns | Low Risk | High Risk |
|  | **Zhang** | Low Risk | Some Concerns | Some Concerns | High Risk | Low Risk | High Risk |
|  | **Alejandra** | Some Concerns | High Risk | Some Concerns | High Risk | Some Concerns | High Risk |
|  | **Chang** | Low Risk | Some Concerns | Low Risk | Some Concerns | Low Risk | Some Concerns |
|  | **Oei** | Low Risk | High Risk | Low Risk | Some Concerns | Low Risk | High Risk |
|  | **Schad** | Low Risk | Some Concerns | Some Concerns | High Risk | Low Risk | High Risk |
|  | **Leite** | Low Risk | High Risk | Low Risk | Some Concerns | Low Risk | High Risk |
|  | **Barcelos** | Low Risk | Some Concerns | Low Risk | High Risk | Low Risk | High Risk |
|  | **Bertoli** | Low Risk | High Risk | Low Risk | Some Concerns | Low Risk | High Risk |
|  | **Silveira** | Some Concerns | High Risk | Some Concerns | Some Concerns | Low Risk | High Risk |
|  | **Lam** | Low Risk | Some Concerns | Low Risk | Some Concerns | Low Risk | Some Concerns |
|  | **Larkey** | Low Risk | Some Concerns | Low Risk | High Risk | Low Risk | High Risk |
|  | **Petzold** | Low Risk | High Risk | Low Risk | Some Concerns | Low Risk | High Risk |
|  | **Yang** | Low Risk | Some Concerns | Low Risk | Some Concerns | Low Risk | Some Concerns |
|  | **Bertoli** | Low Risk | High Risk | Some Concerns | Some Concerns | Low Risk | High Risk |
|  | **Hülya** | Some Concerns | High Risk | Low Risk | Some Concerns | Some Concerns | High Risk |
|  | **Boing** | Low Risk | Some Concerns | Low Risk | High Risk | Low Risk | High Risk |
|  | **Boing** | Low Risk | High Risk | Low Risk | Some Concerns | Low Risk | High Risk |
|  | **Thongteratham** | Low Risk | Some Concerns | Some Concerns | High Risk | Low Risk | High Risk |
|  | **Yao** | Low Risk | High Risk | Low Risk | Some Concerns | Low Risk | High Risk |

**
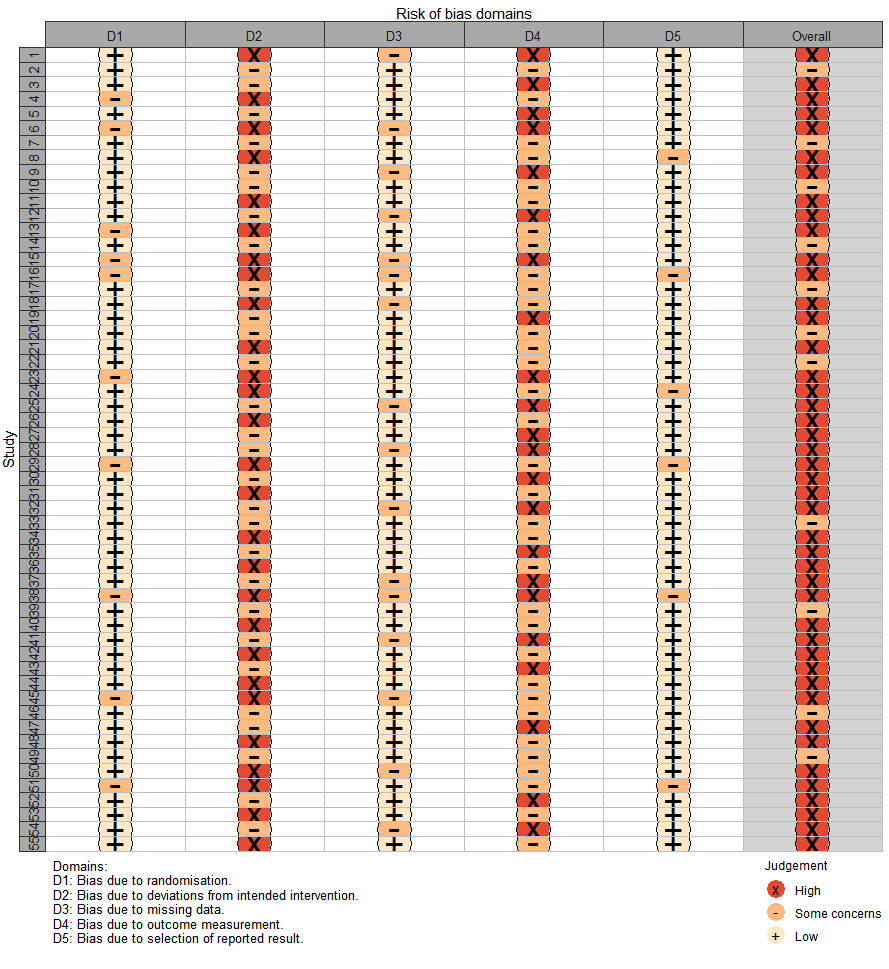
**

**Figure S4. Risk of bias assessment for each included study using the Cochrane Risk of Bias 2.0 tool. D1 = bias due to randomization; D2 = bias due to deviations from intended intervention; D3 = bias due to missing data; D4 = bias due to outcome measurement; D5 = bias due to selection of reported result.**

## Supplementary Table S4. GRADE evidence profile for pairwise meta-analysis results.

| **Outcome** | **Comparison** | **Hedges' g**  **(95% CI)** | **k** | **N** | **I²** | **Risk of Bias** | **Inconsistency** | **Imprecision** | **Indirectness** | **Publication Bias** | **GRADE** |
| --- | --- | --- | --- | --- | --- | --- | --- | --- | --- | --- | --- |
| **Anxiety** | TC vs CON | −0.20 (−0.46, 0.07) | 2 | 215 | 0.0% | ↓1 | Not downgraded | ↓1 | Not downgraded | Not downgraded | ⊕⊕⊖⊖ Low |
| **Anxiety** | QG vs CON | −0.49 (−0.97, −0.02) | 1 | 70 | 0.0% | ↓1 | Not downgraded | Not downgraded | Not downgraded | Not downgraded | ⊕⊕⊕⊖ Moderate |
| **Anxiety** | Pilates vs CON | −0.68 (−1.14, −0.22) | 2 | 103 | 24.0% | ↓1 | Not downgraded | ↓1 | Not downgraded | ↓1 | ⊕⊖⊖⊖ Very Low |
| **Anxiety** | Yoga vs CON | −0.81 (−1.74, 0.12) | 9 | 847 | 97.4% | ↓1 | ↓1 | Not downgraded | Not downgraded | Not downgraded | ⊕⊕⊖⊖ Low |
| **Anxiety** | DC vs CON | −1.31 (−2.40, −0.21) | 4 | 203 | 86.7% | ↓1 | ↓1 | Not downgraded | Not downgraded | Not downgraded | ⊕⊕⊖⊖ Low |
| **QoL** | Yoga vs Pilates | 0.89 (−0.66, 2.44) | 2 | 104 | 92.1% | ↓1 | ↓1 | ↓1 | Not downgraded | Not downgraded | ⊕⊖⊖⊖ Very Low |
| **QoL** | QG vs CON | 0.48 (−0.42, 1.38) | 2 | 164 | 87.4% | ↓1 | ↓1 | Not downgraded | Not downgraded | Not downgraded | ⊕⊕⊕⊖ Moderate |
| **QoL** | DC vs CON | 0.47 (0.04, 0.90) | 3 | 353 | 70.4% | ↓1 | Not downgraded | Not downgraded | Not downgraded | ↓1 | ⊕⊖⊖⊖ Very Low |
| **QoL** | Yoga vs CON | 0.45 (0.04, 0.86) | 23 | 1690 | 93.8% | ↓1 | ↓1 | Not downgraded | Not downgraded | Not downgraded | ⊕⊕⊖⊖ Low |
| **QoL** | TC vs CON | 0.41 (−0.22, 1.04) | 2 | 40 | 0.0% | ↓1 | Not downgraded | ↓1 | Not downgraded | ↓1 | ⊕⊖⊖⊖ Very Low |
| **QoL** | Pilates vs CON | 0.24 (−0.39, 0.87) | 1 | 42 | 0.0% | ↓1 | Not downgraded | Not downgraded | Not downgraded | Not downgraded | ⊕⊕⊕⊖ Moderate |
| **QoL** | Pilates vs Yoga | 0.19 (−0.31, 0.70) | 1 | 60 | 0.0% | ↓1 | Not downgraded | Not downgraded | Not downgraded | Not downgraded | ⊕⊕⊕⊖ Moderate |
| **Depression** | DC vs CON | −0.19 (−0.66, 0.29) | 6 | 286 | 69.0% | ↓1 | ↓1 | ↓1 | Not downgraded | Not downgraded | ⊕⊖⊖⊖ Very Low |
| **Depression** | TC vs CON | −0.28 (−0.84, 0.27) | 3 | 315 | 82.6% | ↓1 | ↓1 | ↓1 | Not downgraded | Not downgraded | ⊕⊖⊖⊖ Very Low |
| **Depression** | Pilates vs CON | −0.29 (−0.67, 0.09) | 6 | 236 | 52.5% | ↓1 | Not downgraded | Not downgraded | Not downgraded | Not downgraded | ⊕⊕⊕⊖ Moderate |
| **Depression** | QG vs CON | −0.35 (−1.02, 0.32) | 2 | 166 | 78.0% | ↓1 | ↓1 | Not downgraded | Not downgraded | Not downgraded | ⊕⊕⊖⊖ Low |
| **Depression** | Yoga vs CON | −0.40 (−0.89, 0.09) | 16 | 1485 | 95.1% | ↓1 | ↓1 | Not downgraded | Not downgraded | Not downgraded | ⊕⊕⊖⊖ Low |

Note: Evidence quality was assessed using the GRADE framework, starting at high (⊕⊕⊕⊕) for RCTs. ↓1 = downgraded one level. Risk of bias was downgraded based on RoB 2 judgments; inconsistency was downgraded when I² > 75%; imprecision was downgraded when the 95% CI crossed the null effect with small sample size; publication bias was downgraded based on funnel plot asymmetry. k = number of studies; N = total participants; QoL = quality of life; CON = control; DC = Dance; TC = Tai Chi; QG = Qigong.

# **Supplementary File 4 Network Meta-Analysis**

**Quality of Life**


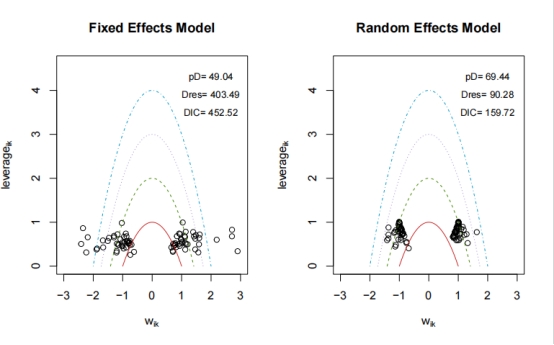


**fixed-effects versus random-effects models.**


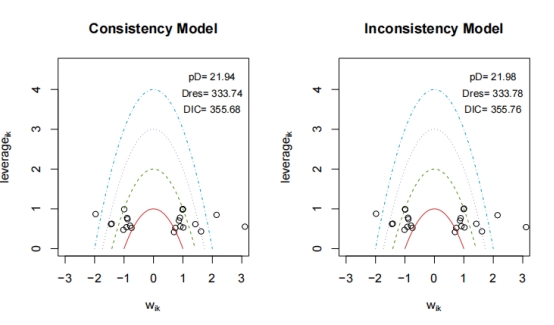


**consistency versus inconsistency models.**

**
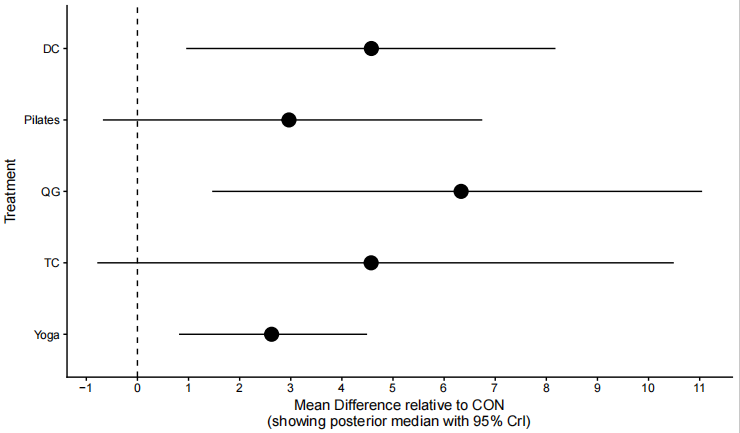
**

**Figure S5. Forest plot of network meta-analysis results for quality of life**


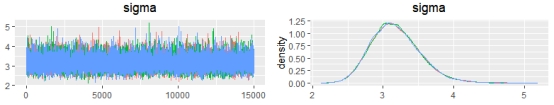


**trace plot (left) and density plot (right) of the between-study heterogeneity parameter (sigma).**

**Depression**


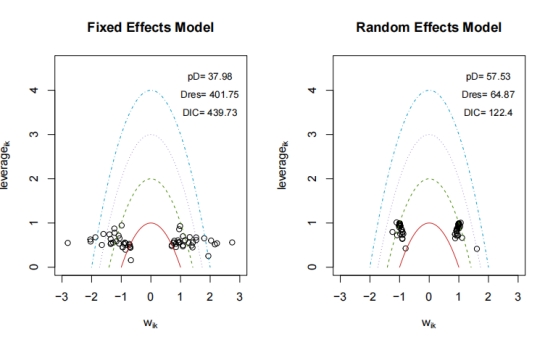


**fixed-effects versus random-effects models.**


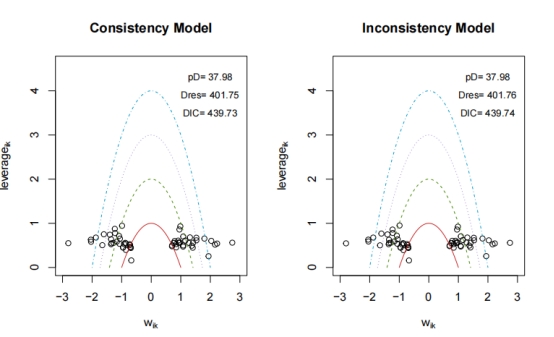


**consistency versus inconsistency models.**

**
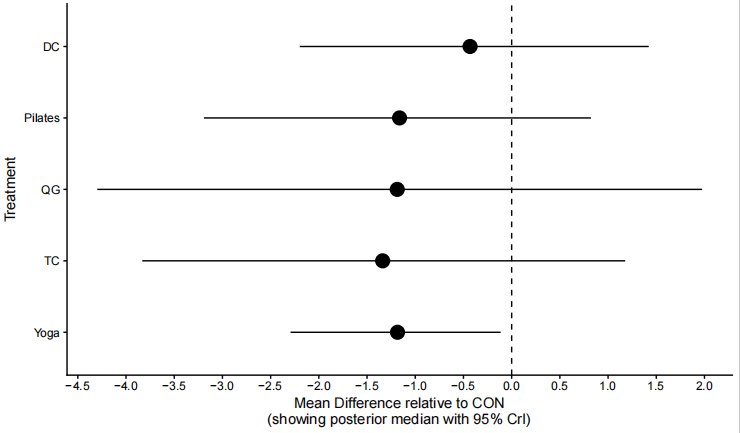
**

**Figure S5. Forest plot of network meta-analysis results for quality of life**


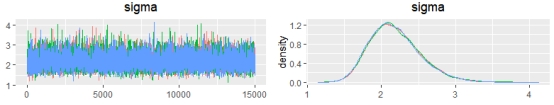


**trace plot (left) and density plot (right) of the between-study heterogeneity parameter (sigma).**

**Anxiety**


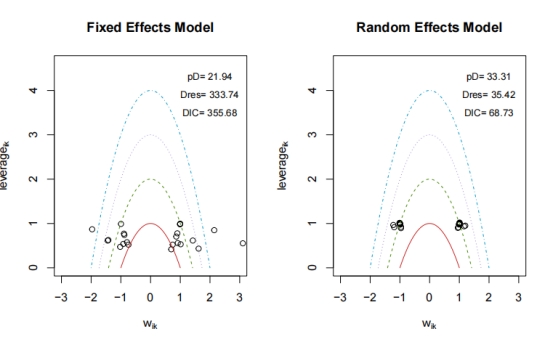


**fixed-effects versus random-effects models.**


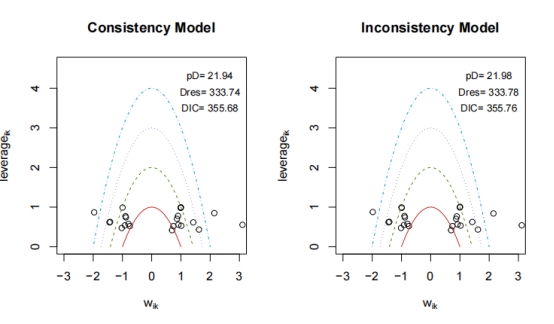


**consistency versus inconsistency models.**

**
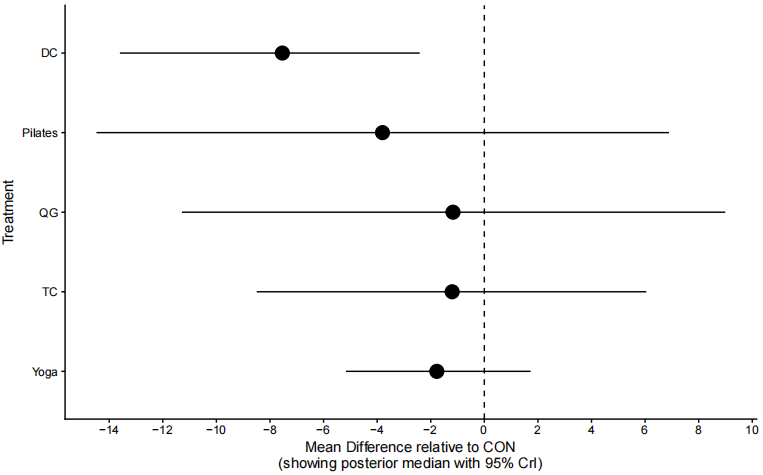
**

**Figure S5. Forest plot of network meta-analysis results for quality of life**


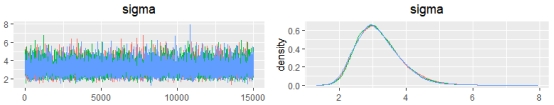


**trace plot (left) and density plot (right) of the between-study heterogeneity parameter (sigma).**
